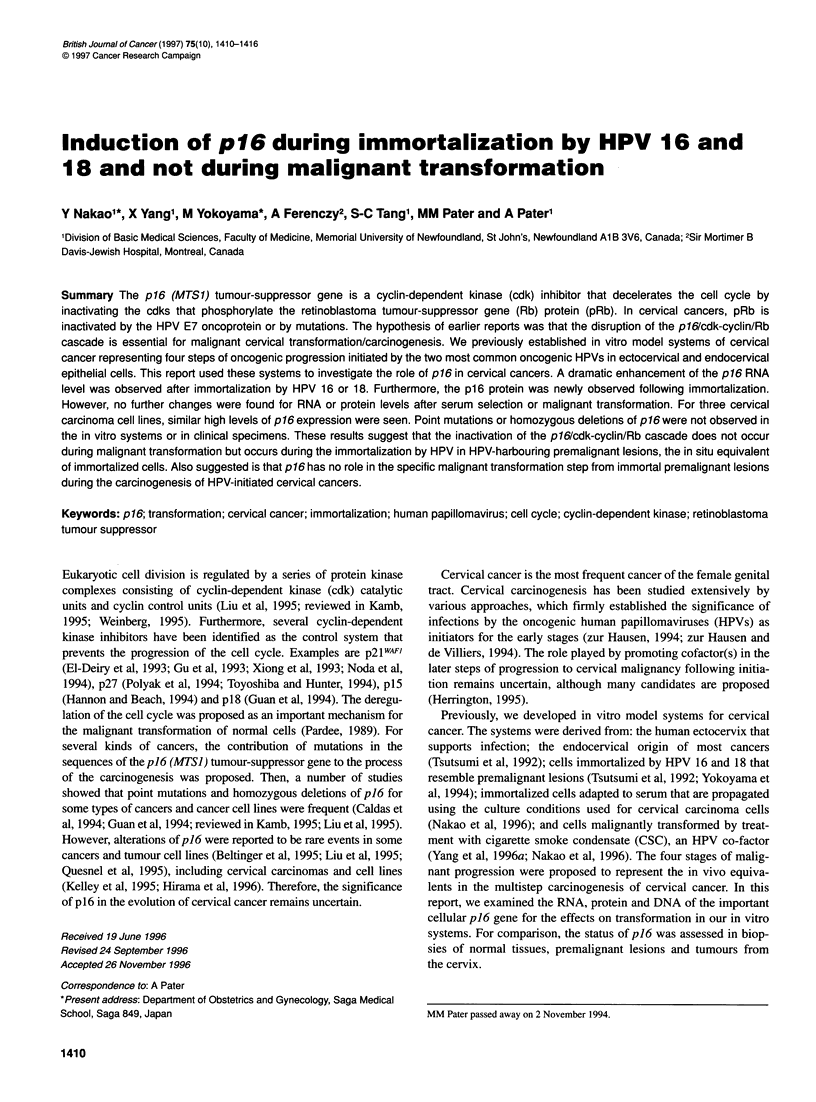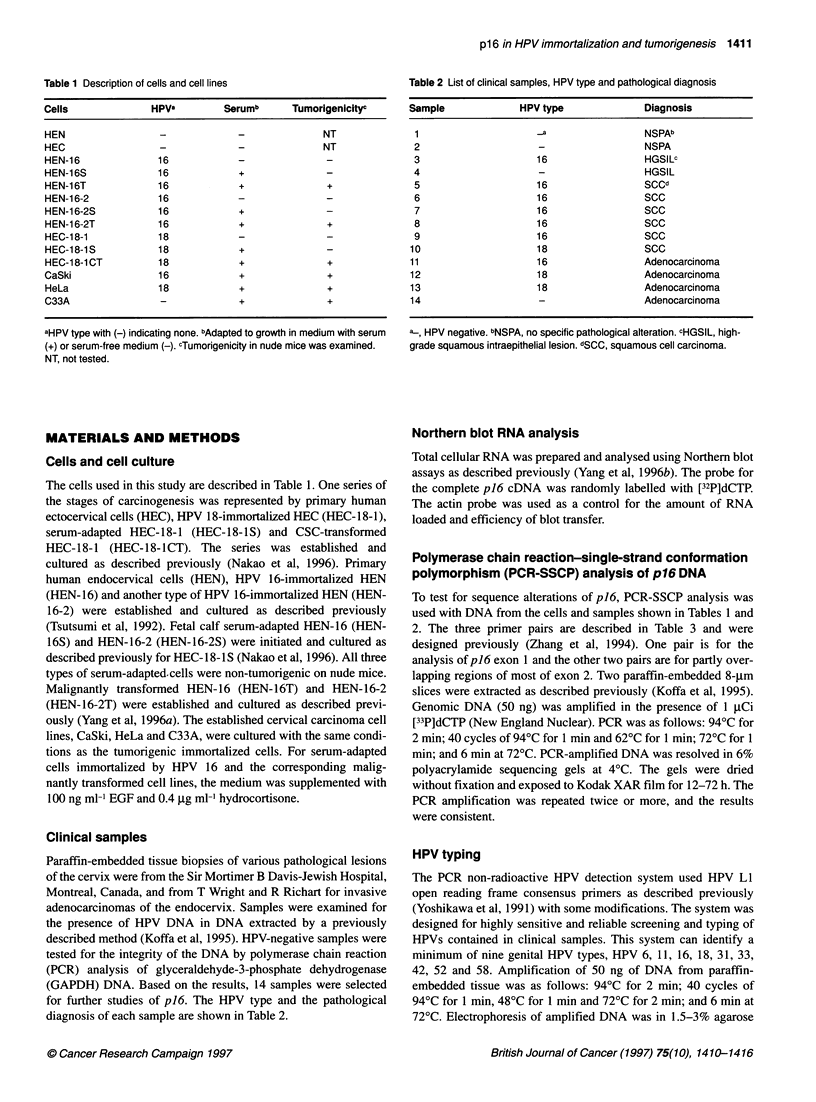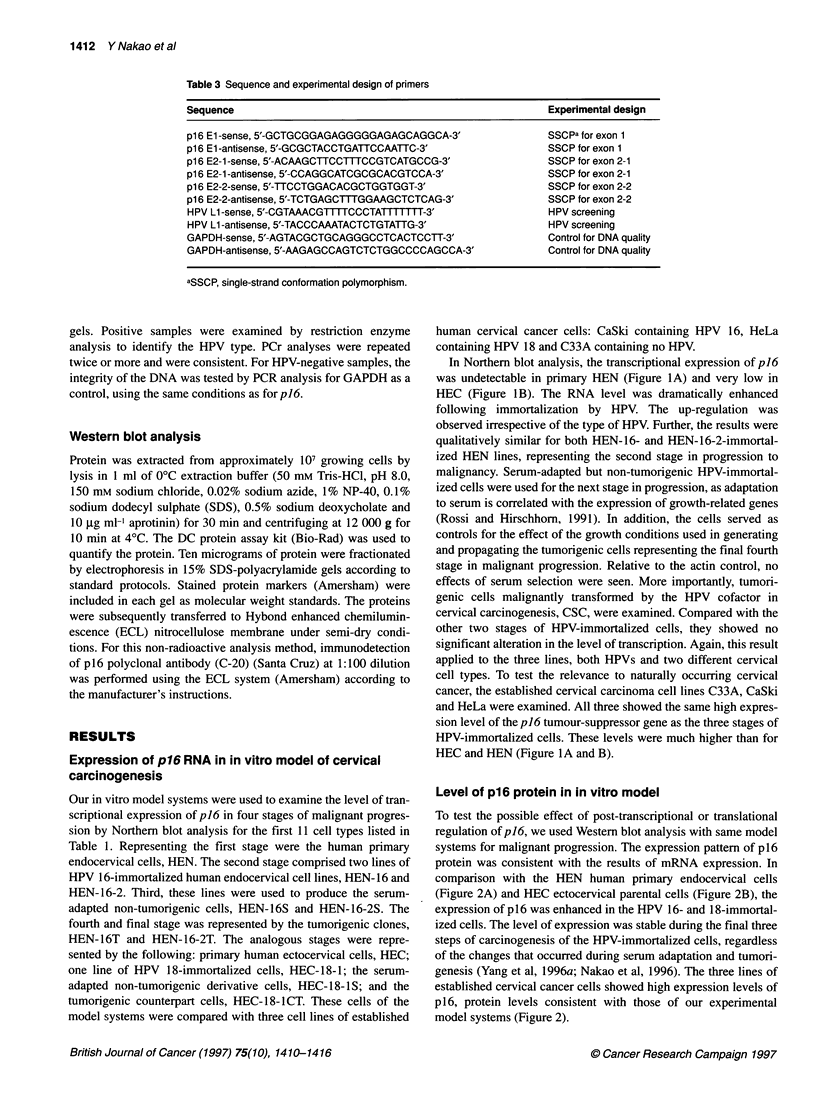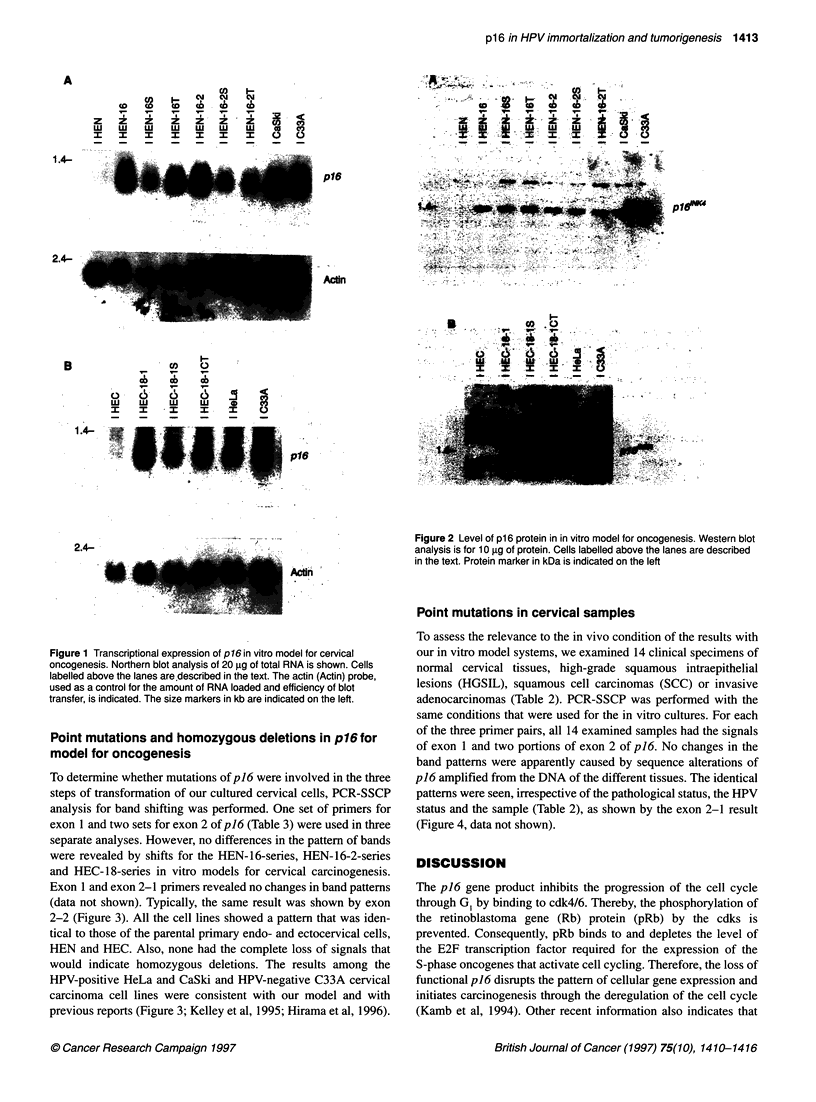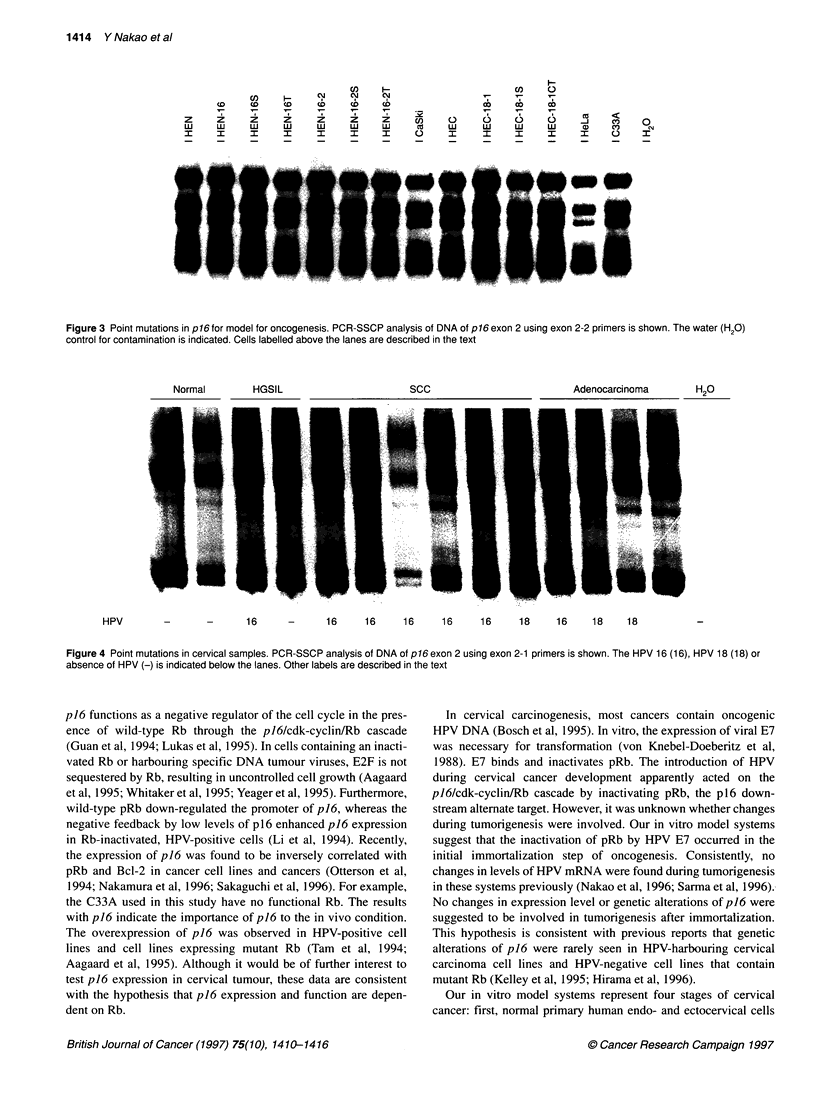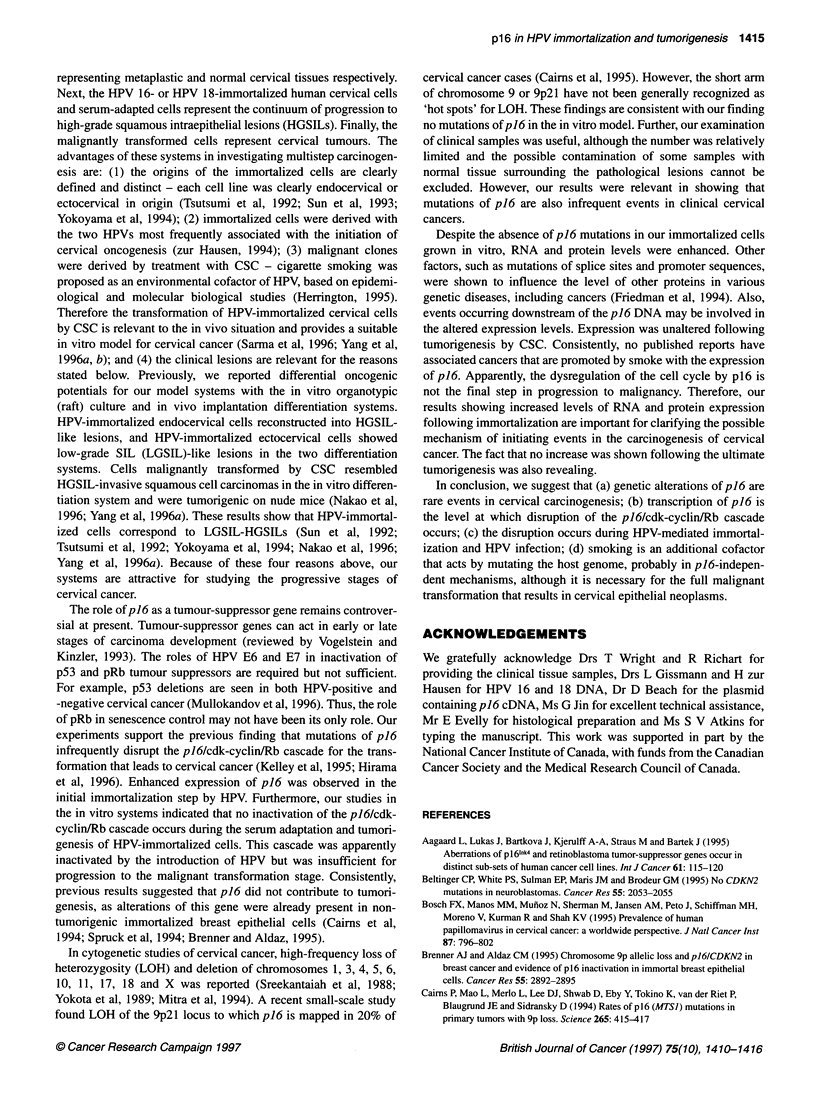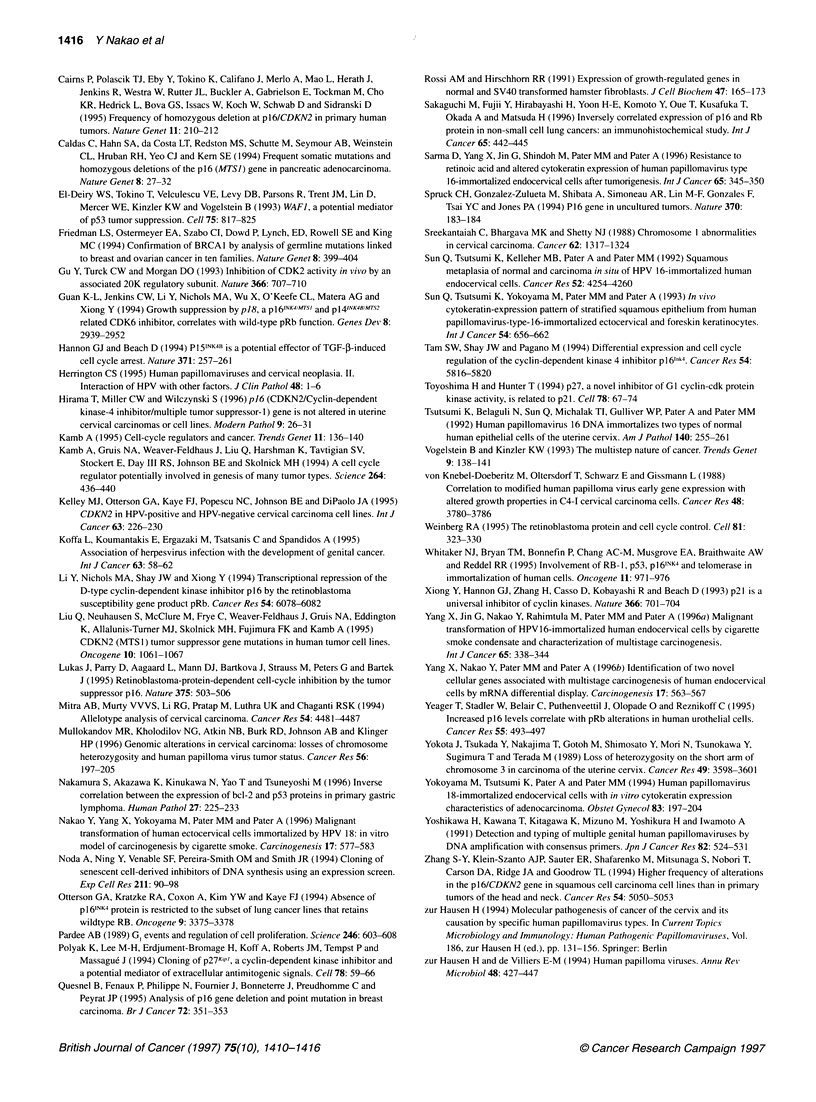# Induction of p16 during immortalization by HPV 16 and 18 and not during malignant transformation.

**DOI:** 10.1038/bjc.1997.243

**Published:** 1997

**Authors:** Y. Nakao, X. Yang, M. Yokoyama, A. Ferenczy, S. C. Tang, M. M. Pater, A. Pater

**Affiliations:** Division of Basic Medical Sciences, Faculty of Medicine, Memorial University of Newfoundland, St John's, Canada.

## Abstract

**Images:**


British Journal of Cancer (1997) 75(10), 1410–1416
© 1997 Cancer Research Campaign

### Induction of *p16* during immortalization by HPV 16 and 18 and not during malignant transformation

**Y Nakao[1]*, X Yang[1], M Yokoyama*, A Ferenczy[2], S-C Tang[1], MM Pater and A Pater[1]**

[1]Division of Basic Medical Sciences, Faculty of Medicine, Memorial University of Newfoundland, St John's, Newfoundland A1B 3V6, Canada; [2]Sir Mortimer B Davis-Jewish Hospital, Montreal, Canada

**Summary** The *p16 (MTS1)* tumour-suppressor gene is a cyclin-dependent kinase (cdk) inhibitor that decelerates the cell cycle by inactivating the cdks that phosphorylate the retinoblastoma tumour-suppressor gene (Rb) protein (pRb). In cervical cancers, pRb is inactivated by the HPV E7 oncoprotein or by mutations. The hypothesis of earlier reports was that the disruption of the *p16*/cdk-cyclin/Rb cascade is essential for malignant cervical transformation/carcinogenesis. We previously established in vitro model systems of cervical cancer representing four steps of oncogenic progression initiated by the two most common oncogenic HPVs in ectocervical and endocervical epithelial cells. This report used these systems to investigate the role of *p16* in cervical cancers. A dramatic enhancement of the *p16* RNA level was observed after immortalization by HPV 16 or 18. Furthermore, the p16 protein was newly observed following immortalization. However, no further changes were found for RNA or protein levels after serum selection or malignant transformation. For three cervical carcinoma cell lines, similar high levels of *p16* expression were seen. Point mutations or homozygous deletions of *p16* were not observed in the in vitro systems or in clinical specimens. These results suggest that the inactivation of the *p16*/cdk-cyclin/Rb cascade does not occur during malignant transformation but occurs during the immortalization by HPV in HPV-harbouring premalignant lesions, the in situ equivalent of immortalized cells. Also suggested is that *p16* has no role in the specific malignant transformation step from immortal premalignant lesions during the carcinogenesis of HPV-initiated cervical cancers.

**Keywords:** *p16*; transformation; cervical cancer; immortalization; human papillomavirus; cell cycle; cyclin-dependent kinase; retinoblastoma tumour suppressor

Eukaryotic cell division is regulated by a series of protein kinase complexes consisting of cyclin-dependent kinase (cdk) catalytic units and cyclin control units (Liu et al, 1995; reviewed in Kamb, 1995; Weinberg, 1995). Furthermore, several cyclin-dependent kinase inhibitors have been identified as the control system that prevents the progression of the cell cycle. Examples are p21$^{WAF1}$ (El-Deiry et al, 1993; Gu et al, 1993; Xiong et al, 1993; Noda et al, 1994), p27 (Polyak et al, 1994; Toyoshiba and Hunter, 1994), p15 (Hannon and Beach, 1994) and p18 (Guan et al, 1994). The deregulation of the cell cycle was proposed as an important mechanism for the malignant transformation of normal cells (Pardee, 1989). For several kinds of cancers, the contribution of mutations in the sequences of the *p16 (MTS1)* tumour-suppressor gene to the process of the carcinogenesis was proposed. Then, a number of studies showed that point mutations and homozygous deletions of *p16* for some types of cancers and cancer cell lines were frequent (Caldas et al, 1994; Guan et al, 1994; reviewed in Kamb, 1995; Liu et al, 1995). However, alterations of *p16* were reported to be rare events in some cancers and tumour cell lines (Beltinger et al, 1995; Liu et al, 1995; Quesnel et al, 1995), including cervical carcinomas and cell lines (Kelley et al, 1995; Hirama et al, 1996). Therefore, the significance of p16 in the evolution of cervical cancer remains uncertain.

*Received 19 June 1996*
*Revised 24 September 1996*
*Accepted 26 November 1996*

*Correspondence to*: A Pater
**Present address*: Department of Obstetrics and Gynecology, Saga Medical School, Saga 849, Japan

Cervical cancer is the most frequent cancer of the female genital tract. Cervical carcinogenesis has been studied extensively by various approaches, which firmly established the significance of infections by the oncogenic human papillomaviruses (HPVs) as initiators for the early stages (zur Hausen, 1994; zur Hausen and de Villiers, 1994). The role played by promoting cofactor(s) in the later steps of progression to cervical malignancy following initiation remains uncertain, although many candidates are proposed (Herrington, 1995).

Previously, we developed in vitro model systems for cervical cancer. The systems were derived from: the human ectocervix that supports infection; the endocervical origin of most cancers (Tsutsumi et al, 1992); cells immortalized by HPV 16 and 18 that resemble premalignant lesions (Tsutsumi et al, 1992; Yokoyama et al, 1994); immortalized cells adapted to serum that are propagated using the culture conditions used for cervical carcinoma cells (Nakao et al, 1996); and cells malignantly transformed by treatment with cigarette smoke condensate (CSC), an HPV co-factor (Yang et al, 1996*a*; Nakao et al, 1996). The four stages of malignant progression were proposed to represent the in vivo equivalents in the multistep carcinogenesis of cervical cancer. In this report, we examined the RNA, protein and DNA of the important cellular *p16* gene for the effects on transformation in our in vitro systems. For comparison, the status of *p16* was assessed in biopsies of normal tissues, premalignant lesions and tumours from the cervix.

MM Pater passed away on 2 November 1994.

**Table 1** Description of cells and cell lines

| Cells | HPV[a] | Serum[b] | Tumorigenicity[c] |
|---|---|---|---|
| HEN | – | – | NT |
| HEC | – | – | NT |
| HEN-16 | 16 | – | – |
| HEN-16S | 16 | + | – |
| HEN-16T | 16 | + | + |
| HEN-16-2 | 16 | – | – |
| HEN-16-2S | 16 | + | – |
| HEN-16-2T | 16 | + | + |
| HEC-18-1 | 18 | – | – |
| HEC-18-1S | 18 | + | – |
| HEC-18-1CT | 18 | + | + |
| CaSki | 16 | + | + |
| HeLa | 18 | + | + |
| C33A | – | + | + |

[a]HPV type with (–) indicating none. [b]Adapted to growth in medium with serum (+) or serum-free medium (–). [c]Tumorigenicity in nude mice was examined. NT, not tested.

**Table 2** List of clinical samples, HPV type and pathological diagnosis

| Sample | HPV type | Diagnosis |
|---|---|---|
| 1 | –[a] | NSPA[b] |
| 2 | – | NSPA |
| 3 | 16 | HGSIL[c] |
| 4 | – | HGSIL |
| 5 | 16 | SCC[d] |
| 6 | 16 | SCC |
| 7 | 16 | SCC |
| 8 | 16 | SCC |
| 9 | 16 | SCC |
| 10 | 18 | SCC |
| 11 | 16 | Adenocarcinoma |
| 12 | 18 | Adenocarcinoma |
| 13 | 18 | Adenocarcinoma |
| 14 | – | Adenocarcinoma |

[a]–, HPV negative. [b]NSPA, no specific pathological alteration. [c]HGSIL, high-grade squamous intraepithelial lesion. [d]SCC, squamous cell carcinoma.

### MATERIALS AND METHODS

### Cells and cell culture

The cells used in this study are described in Table 1. One series of the stages of carcinogenesis was represented by primary human ectocervical cells (HEC), HPV 18-immortalized HEC (HEC-18-1), serum-adapted HEC-18-1 (HEC-18-1S) and CSC-transformed HEC-18-1 (HEC-18-1CT). The series was established and cultured as described previously (Nakao et al, 1996). Primary human endocervical cells (HEN), HPV 16-immortalized HEN (HEN-16) and another type of HPV 16-immortalized HEN (HEN-16-2) were established and cultured as described previously (Tsutsumi et al, 1992). Fetal calf serum-adapted HEN-16 (HEN-16S) and HEN-16-2 (HEN-16-2S) were initiated and cultured as described previously for HEC-18-1S (Nakao et al, 1996). All three types of serum-adapted cells were non-tumorigenic on nude mice. Malignantly transformed HEN-16 (HEN-16T) and HEN-16-2 (HEN-16-2T) were established and cultured as described previously (Yang et al, 1996*a*). The established cervical carcinoma cell lines, CaSki, HeLa and C33A, were cultured with the same conditions as the tumorigenic immortalized cells. For serum-adapted cells immortalized by HPV 16 and the corresponding malignantly transformed cell lines, the medium was supplemented with 100 ng $ml^{-1}$ EGF and 0.4 µg $ml^{-1}$ hydrocortisone.

### Clinical samples

Paraffin-embedded tissue biopsies of various pathological lesions of the cervix were from the Sir Mortimer B Davis-Jewish Hospital, Montreal, Canada, and from T Wright and R Richart for invasive adenocarcinomas of the endocervix. Samples were examined for the presence of HPV DNA in DNA extracted by a previously described method (Koffa et al, 1995). HPV-negative samples were tested for the integrity of the DNA by polymerase chain reaction (PCR) analysis of glyceraldehyde-3-phosphate dehydrogenase (GAPDH) DNA. Based on the results, 14 samples were selected for further studies of *p16*. The HPV type and the pathological diagnosis of each sample are shown in Table 2.

### Northern blot RNA analysis

Total cellular RNA was prepared and analysed using Northern blot assays as described previously (Yang et al, 1996*b*). The probe for the complete *p16* cDNA was randomly labelled with $[^{32}P]$dCTP. The actin probe was used as a control for the amount of RNA loaded and efficiency of blot transfer.

### Polymerase chain reaction–single-strand conformation polymorphism (PCR-SSCP) analysis of *p16* DNA

To test for sequence alterations of *p16*, PCR-SSCP analysis was used with DNA from the cells and samples shown in Tables 1 and 2. The three primer pairs are described in Table 3 and were designed previously (Zhang et al, 1994). One pair is for the analysis of *p16* exon 1 and the other two pairs are for partly overlapping regions of most of exon 2. Two paraffin-embedded 8-µm slices were extracted as described previously (Koffa et al, 1995). Genomic DNA (50 ng) was amplified in the presence of 1 µCi $[^{33}P]$dCTP (New England Nuclear). PCR was as follows: 94°C for 2 min; 40 cycles of 94°C for 1 min and 62°C for 1 min; 72°C for 1 min; and 6 min at 72°C. PCR-amplified DNA was resolved in 6% polyacrylamide sequencing gels at 4°C. The gels were dried without fixation and exposed to Kodak XAR film for 12–72 h. The PCR amplification was repeated twice or more, and the results were consistent.

### HPV typing

The PCR non-radioactive HPV detection system used HPV L1 open reading frame consensus primers as described previously (Yoshikawa et al, 1991) with some modifications. The system was designed for highly sensitive and reliable screening and typing of HPVs contained in clinical samples. This system can identify a minimum of nine genital HPV types, HPV 6, 11, 16, 18, 31, 33, 42, 52 and 58. Amplification of 50 ng of DNA from paraffin-embedded tissue was as follows: 94°C for 2 min; 40 cycles of 94°C for 1 min, 48°C for 1 min and 72°C for 2 min; and 6 min at 72°C. Electrophoresis of amplified DNA was in 1.5–3% agarose

© Cancer Research Campaign 1997

**Table 3** Sequence and experimental design of primers

| Sequence | Experimental design |
|---|---|
| p16 E1-sense, 5′-GCTGCGGAGAGGGGGAGAGCAGGCA-3′ | SSCP[a] for exon 1 |
| p16 E1-antisense, 5′-GCGCTACCTGATTCCAATTC-3′ | SSCP for exon 1 |
| p16 E2-1-sense, 5′-ACAAGCTTCCTTTCCGTCATGCCG-3′ | SSCP for exon 2-1 |
| p16 E2-1-antisense, 5′-CCAGGCATCGCGCACGTCCA-3′ | SSCP for exon 2-1 |
| p16 E2-2-sense, 5′-TTCCTGGACACGCTGGTGGT-3′ | SSCP for exon 2-2 |
| p16 E2-2-antisense, 5′-TCTGAGCTTTGGAAGCTCTCAG-3′ | SSCP for exon 2-2 |
| HPV L1-sense, 5′-CGTAAACGTTTTCCCTATTTTTTT-3′ | HPV screening |
| HPV L1-antisense, 5′-TACCCAAATACTCTGTATTG-3′ | HPV screening |
| GAPDH-sense, 5′-AGTACGCTGCAGGGCCTCACTCCTT-3′ | Control for DNA quality |
| GAPDH-antisense, 5′-AAGAGCCAGTCTCTGGCCCCAGCCA-3′ | Control for DNA quality |

[a]SSCP, single-strand conformation polymorphism.

gels. Positive samples were examined by restriction enzyme analysis to identify the HPV type. PCr analyses were repeated twice or more and were consistent. For HPV-negative samples, the integrity of the DNA was tested by PCR analysis for GAPDH as a control, using the same conditions as for *p16*.

### Western blot analysis

Protein was extracted from approximately $10^7$ growing cells by lysis in 1 ml of 0°C extraction buffer (50 mM Tris-HCl, pH 8.0, 150 mM sodium chloride, 0.02% sodium azide, 1% NP-40, 0.1% sodium dodecyl sulphate (SDS), 0.5% sodium deoxycholate and 10 $\mu$g $ml^{-1}$ aprotinin) for 30 min and centrifuging at 12 000 **g** for 10 min at 4°C. The DC protein assay kit (Bio-Rad) was used to quantify the protein. Ten micrograms of protein were fractionated by electrophoresis in 15% SDS-polyacrylamide gels according to standard protocols. Stained protein markers (Amersham) were included in each gel as molecular weight standards. The proteins were subsequently transferred to Hybond enhanced chemiluminescence (ECL) nitrocellulose membrane under semi-dry conditions. For this non-radioactive analysis method, immunodetection of p16 polyclonal antibody (C-20) (Santa Cruz) at 1:100 dilution was performed using the ECL system (Amersham) according to the manufacturer's instructions.

### RESULTS

### Expression of *p16* RNA in in vitro model of cervical carcinogenesis

Our in vitro model systems were used to examine the level of transcriptional expression of *p16* in four stages of malignant progression by Northern blot analysis for the first 11 cell types listed in Table 1. Representing the first stage were the human primary endocervical cells, HEN. The second stage comprised two lines of HPV 16-immortalized human endocervical cell lines, HEN-16 and HEN-16-2. Third, these lines were used to produce the serum-adapted non-tumorigenic cells, HEN-16S and HEN-16-2S. The fourth and final stage was represented by the tumorigenic clones, HEN-16T and HEN-16-2T. The analogous stages were represented by the following: primary human ectocervical cells, HEC; one line of HPV 18-immortalized cells, HEC-18-1; the serum-adapted non-tumorigenic derivative cells, HEC-18-1S; and the tumorigenic counterpart cells, HEC-18-1CT. These cells of the model systems were compared with three cell lines of established human cervical cancer cells: CaSki containing HPV 16, HeLa containing HPV 18 and C33A containing no HPV.

In Northern blot analysis, the transcriptional expression of *p16* was undetectable in primary HEN (Figure 1A) and very low in HEC (Figure 1B). The RNA level was dramatically enhanced following immortalization by HPV. The up-regulation was observed irrespective of the type of HPV. Further, the results were qualitatively similar for both HEN-16- and HEN-16-2-immortalized HEN lines, representing the second stage in progression to malignancy. Serum-adapted but non-tumorigenic HPV-immortalized cells were used for the next stage in progression, as adaptation to serum is correlated with the expression of growth-related genes (Rossi and Hirschhorn, 1991). In addition, the cells served as controls for the effect of the growth conditions used in generating and propagating the tumorigenic cells representing the final fourth stage in malignant progression. Relative to the actin control, no effects of serum selection were seen. More importantly, tumorigenic cells malignantly transformed by the HPV cofactor in cervical carcinogenesis, CSC, were examined. Compared with the other two stages of HPV-immortalized cells, they showed no significant alteration in the level of transcription. Again, this result applied to the three lines, both HPVs and two different cervical cell types. To test the relevance to naturally occurring cervical cancer, the established cervical carcinoma cell lines C33A, CaSki and HeLa were examined. All three showed the same high expression level of the *p16* tumour-suppressor gene as the three stages of HPV-immortalized cells. These levels were much higher than for HEC and HEN (Figure 1A and B).

### Level of p16 protein in in vitro model

To test the possible effect of post-transcriptional or translational regulation of *p16*, we used Western blot analysis with same model systems for malignant progression. The expression pattern of p16 protein was consistent with the results of mRNA expression. In comparison with the HEN human primary endocervical cells (Figure 2A) and HEC ectocervical parental cells (Figure 2B), the expression of p16 was enhanced in the HPV 16- and 18-immortalized cells. The level of expression was stable during the final three steps of carcinogenesis of the HPV-immortalized cells, regardless of the changes that occurred during serum adaptation and tumorigenesis (Yang et al, 1996*a*; Nakao et al, 1996). The three lines of established cervical cancer cells showed high expression levels of p16, protein levels consistent with those of our experimental model systems (Figure 2).

© Cancer Research Campaign 1997

**Figure 1** Transcriptional expression of *p16* in vitro model for cervical oncogenesis. Northern blot analysis of 20 µg of total RNA is shown. Cells labelled above the lanes are described in the text. The actin (Actin) probe, used as a control for the amount of RNA loaded and efficiency of blot transfer, is indicated. The size markers in kb are indicated on the left.

### Point mutations and homozygous deletions in *p16* for model for oncogenesis

To determine whether mutations of *p16* were involved in the three steps of transformation of our cultured cervical cells, PCR-SSCP analysis for band shifting was performed. One set of primers for exon 1 and two sets for exon 2 of *p16* (Table 3) were used in three separate analyses. However, no differences in the pattern of bands were revealed by shifts for the HEN-16-series, HEN-16-2-series and HEC-18-series in vitro models for cervical carcinogenesis. Exon 1 and exon 2–1 primers revealed no changes in band patterns (data not shown). Typically, the same result was shown by exon 2–2 (Figure 3). All the cell lines showed a pattern that was identical to those of the parental primary endo- and ectocervical cells, HEN and HEC. Also, none had the complete loss of signals that would indicate homozygous deletions. The results among the HPV-positive HeLa and CaSki and HPV-negative C33A cervical carcinoma cell lines were consistent with our model and with previous reports (Figure 3; Kelley et al, 1995; Hirama et al, 1996).

**Figure 2** Level of p16 protein in in vitro model for oncogenesis. Western blot analysis is for 10 µg of protein. Cells labelled above the lanes are described in the text. Protein marker in kDa is indicated on the left

### Point mutations in cervical samples

To assess the relevance to the in vivo condition of the results with our in vitro model systems, we examined 14 clinical specimens of normal cervical tissues, high-grade squamous intraepithelial lesions (HGSIL), squamous cell carcinomas (SCC) or invasive adenocarcinomas (Table 2). PCR-SSCP was performed with the same conditions that were used for the in vitro cultures. For each of the three primer pairs, all 14 examined samples had the signals of exon 1 and two portions of exon 2 of *p16*. No changes in the band patterns were apparently caused by sequence alterations of *p16* amplified from the DNA of the different tissues. The identical patterns were seen, irrespective of the pathological status, the HPV status and the sample (Table 2), as shown by the exon 2–1 result (Figure 4, data not shown).

### DISCUSSION

The *p16* gene product inhibits the progression of the cell cycle through $G_1$ by binding to cdk4/6. Thereby, the phosphorylation of the retinoblastoma gene (Rb) protein (pRb) by the cdks is prevented. Consequently, pRb binds to and depletes the level of the E2F transcription factor required for the expression of the S-phase oncogenes that activate cell cycling. Therefore, the loss of functional *p16* disrupts the pattern of cellular gene expression and initiates carcinogenesis through the deregulation of the cell cycle (Kamb et al, 1994). Other recent information also indicates that

© Cancer Research Campaign 1997 

**Figure 3** Point mutations in *p16* for model for oncogenesis. PCR-SSCP analysis of DNA of *p16* exon 2 using exon 2-2 primers is shown. The water ($H_2O$) control for contamination is indicated. Cells labelled above the lanes are described in the text

**Figure 4** Point mutations in cervical samples. PCR-SSCP analysis of DNA of *p16* exon 2 using exon 2-1 primers is shown. The HPV 16 (16), HPV 18 (18) or absence of HPV (–) is indicated below the lanes. Other labels are described in the text

*p16* functions as a negative regulator of the cell cycle in the presence of wild-type Rb through the *p16*/cdk-cyclin/Rb cascade (Guan et al, 1994; Lukas et al, 1995). In cells containing an inactivated Rb or harbouring specific DNA tumour viruses, E2F is not sequestered by Rb, resulting in uncontrolled cell growth (Aagaard et al, 1995; Whitaker et al, 1995; Yeager et al, 1995). Furthermore, wild-type pRb down-regulated the promoter of *p16*, whereas the negative feedback by low levels of p16 enhanced *p16* expression in Rb-inactivated, HPV-positive cells (Li et al, 1994). Recently, the expression of *p16* was found to be inversely correlated with pRb and Bcl-2 in cancer cell lines and cancers (Otterson et al, 1994; Nakamura et al, 1996; Sakaguchi et al, 1996). For example, the C33A used in this study have no functional Rb. The results with *p16* indicate the importance of *p16* to the in vivo condition. The overexpression of *p16* was observed in HPV-positive cell lines and cell lines expressing mutant Rb (Tam et al, 1994; Aagaard et al, 1995). Although it would be of further interest to test *p16* expression in cervical tumour, these data are consistent with the hypothesis that *p16* expression and function are dependent on Rb.

In cervical carcinogenesis, most cancers contain oncogenic HPV DNA (Bosch et al, 1995). In vitro, the expression of viral E7 was necessary for transformation (von Knebel-Doeberitz et al, 1988). E7 binds and inactivates pRb. The introduction of HPV during cervical cancer development apparently acted on the *p16*/cdk-cyclin/Rb cascade by inactivating pRb, the p16 downstream alternate target. However, it was unknown whether changes during tumorigenesis were involved. Our in vitro model systems suggest that the inactivation of pRb by HPV E7 occurred in the initial immortalization step of oncogenesis. Consistently, no changes in levels of HPV mRNA were found during tumorigenesis in these systems previously (Nakao et al, 1996; Sarma et al, 1996). No changes in expression level or genetic alterations of *p16* were suggested to be involved in tumorigenesis after immortalization. This hypothesis is consistent with previous reports that genetic alterations of *p16* were rarely seen in HPV-harbouring cervical carcinoma cell lines and HPV-negative cell lines that contain mutant Rb (Kelley et al, 1995; Hirama et al, 1996).

Our in vitro model systems represent four stages of cervical cancer: first, normal primary human endo- and ectocervical cells

© Cancer Research Campaign 1997

representing metaplastic and normal cervical tissues respectively. Next, the HPV 16- or HPV 18-immortalized human cervical cells and serum-adapted cells represent the continuum of progression to high-grade squamous intraepithelial lesions (HGSILs). Finally, the malignantly transformed cells represent cervical tumours. The advantages of these systems in investigating multistep carcinogenesis are: (1) the origins of the immortalized cells are clearly defined and distinct – each cell line was clearly endocervical or ectocervical in origin (Tsutsumi et al, 1992; Sun et al, 1993; Yokoyama et al, 1994); (2) immortalized cells were derived with the two HPVs most frequently associated with the initiation of cervical oncogenesis (zur Hausen, 1994); (3) malignant clones were derived by treatment with CSC – cigarette smoking was proposed as an environmental cofactor of HPV, based on epidemiological and molecular biological studies (Herrington, 1995). Therefore the transformation of HPV-immortalized cervical cells by CSC is relevant to the in vivo situation and provides a suitable in vitro model for cervical cancer (Sarma et al, 1996; Yang et al, 1996*a*, *b*); and (4) the clinical lesions are relevant for the reasons stated below. Previously, we reported differential oncogenic potentials for our model systems with the in vitro organotypic (raft) culture and in vivo implantation differentiation systems. HPV-immortalized endocervical cells reconstructed into HGSIL-like lesions, and HPV-immortalized ectocervical cells showed low-grade SIL (LGSIL)-like lesions in the two differentiation systems. Cells malignantly transformed by CSC resembled HGSIL-invasive squamous cell carcinomas in the in vitro differentiation system and were tumorigenic on nude mice (Nakao et al, 1996; Yang et al, 1996*a*). These results show that HPV-immortalized cells correspond to LGSIL-HGSILs (Sun et al, 1992; Tsutsumi et al, 1992; Yokoyama et al, 1994; Nakao et al, 1996; Yang et al, 1996*a*). Because of these four reasons above, our systems are attractive for studying the progressive stages of cervical cancer.

The role of *p16* as a tumour-suppressor gene remains controversial at present. Tumour-suppressor genes can act in early or late stages of carcinoma development (reviewed by Vogelstein and Kinzler, 1993). The roles of HPV E6 and E7 in inactivation of p53 and pRb tumour suppressors are required but not sufficient. For example, p53 deletions are seen in both HPV-positive and -negative cervical cancer (Mullokandov et al, 1996). Thus, the role of pRb in senescence control may not have been its only role. Our experiments support the previous finding that mutations of *p16* infrequently disrupt the *p16*/cdk-cyclin/Rb cascade for the transformation that leads to cervical cancer (Kelley et al, 1995; Hirama et al, 1996). Enhanced expression of *p16* was observed in the initial immortalization step by HPV. Furthermore, our studies in the in vitro systems indicated that no inactivation of the *p16*/cdk-cyclin/Rb cascade occurs during the serum adaptation and tumorigenesis of HPV-immortalized cells. This cascade was apparently inactivated by the introduction of HPV but was insufficient for progression to the malignant transformation stage. Consistently, previous results suggested that *p16* did not contribute to tumorigenesis, as alterations of this gene were already present in non-tumorigenic immortalized breast epithelial cells (Cairns et al, 1994; Spruck et al, 1994; Brenner and Aldaz, 1995).

In cytogenetic studies of cervical cancer, high-frequency loss of heterozygosity (LOH) and deletion of chromosomes 1, 3, 4, 5, 6, 10, 11, 17, 18 and X was reported (Sreekantaiah et al, 1988; Yokota et al, 1989; Mitra et al, 1994). A recent small-scale study found LOH of the 9p21 locus to which *p16* is mapped in 20% of cervical cancer cases (Cairns et al, 1995). However, the short arm of chromosome 9 or 9p21 have not been generally recognized as 'hot spots' for LOH. These findings are consistent with our finding no mutations of *p16* in the in vitro model. Further, our examination of clinical samples was useful, although the number was relatively limited and the possible contamination of some samples with normal tissue surrounding the pathological lesions cannot be excluded. However, our results were relevant in showing that mutations of *p16* are also infrequent events in clinical cervical cancers.

Despite the absence of *p16* mutations in our immortalized cells grown in vitro, RNA and protein levels were enhanced. Other factors, such as mutations of splice sites and promoter sequences, were shown to influence the level of other proteins in various genetic diseases, including cancers (Friedman et al, 1994). Also, events occurring downstream of the *p16* DNA may be involved in the altered expression levels. Expression was unaltered following tumorigenesis by CSC. Consistently, no published reports have associated cancers that are promoted by smoke with the expression of *p16*. Apparently, the dysregulation of the cell cycle by p16 is not the final step in progression to malignancy. Therefore, our results showing increased levels of RNA and protein expression following immortalization are important for clarifying the possible mechanism of initiating events in the carcinogenesis of cervical cancer. The fact that no increase was shown following the ultimate tumorigenesis was also revealing.

In conclusion, we suggest that (a) genetic alterations of *p16* are rare events in cervical carcinogenesis; (b) transcription of *p16* is the level at which disruption of the *p16*/cdk-cyclin/Rb cascade occurs; (c) the disruption occurs during HPV-mediated immortalization and HPV infection; (d) smoking is an additional cofactor that acts by mutating the host genome, probably in *p16*-independent mechanisms, although it is necessary for the full malignant transformation that results in cervical epithelial neoplasms.

### ACKNOWLEDGEMENTS

We gratefully acknowledge Drs T Wright and R Richart for providing the clinical tissue samples, Drs L Gissmann and H zur Hausen for HPV 16 and 18 DNA, Dr D Beach for the plasmid containing *p16* cDNA, Ms G Jin for excellent technical assistance, Mr E Evelly for histological preparation and Ms S V Atkins for typing the manuscript. This work was supported in part by the National Cancer Institute of Canada, with funds from the Canadian Cancer Society and the Medical Research Council of Canada.

### REFERENCES

Aagaard L, Lukas J, Bartkova J, Kjerulff A-A, Straus M and Bartek J (1995) Aberrations of p16$^{Ink4}$ and retinoblastoma tumor-suppressor genes occur in distinct sub-sets of human cancer cell lines. *Int J Cancer* **61**: 115–120

Beltinger CP, White PS, Sulman EP, Maris JM and Brodeur GM (1995) No *CDKN2* mutations in neuroblastomas. *Cancer Res* **55**: 2053–2055

Bosch FX, Manos MM, Muñoz N, Sherman M, Jansen AM, Peto J, Schiffman MH, Moreno V, Kurman R and Shah KV (1995) Prevalence of human papillomavirus in cervical cancer: a worldwide perspective. *J Natl Cancer Inst* **87**: 796–802

Brenner AJ and Aldaz CM (1995) Chromosome 9p allelic loss and *p16/CDKN2* in breast cancer and evidence of p16 inactivation in immortal breast epithelial cells. *Cancer Res* **55**: 2892–2895

Cairns P, Mao L, Merlo L, Lee DJ, Shwab D, Eby Y, Tokino K, van der Riet P, Blaugrund JE and Sidransky D (1994) Rates of p16 (*MTS1*) mutations in primary tumors with 9p loss. *Science* **265**: 415–417

Cairns P, Polascik TJ, Eby Y, Tokino K, Califano J, Merlo A, Mao L, Herath J, Jenkins R, Westra W, Rutter JL, Buckler A, Gabrielson E, Tockman M, Cho KR, Hedrick L, Bova GS, Issacs W, Koch W, Schwab D and Sidranski D (1995) Frequency of homozygous deletion at p16/*CDKN2* in primary human tumors. *Nature Genet* **11**: 210–212

Caldas C, Hahn SA, da Costa LT, Redston MS, Schutte M, Seymour AB, Weinstein CL, Hruban RH, Yeo CJ and Kern SE (1994) Frequent somatic mutations and homozygous deletions of the p16 (*MTS1*) gene in pancreatic adenocarcinoma. *Nature Genet* **8**: 27–32

El-Deiry WS, Tokino T, Velculescu VE, Levy DB, Parsons R, Trent JM, Lin D, Mercer WE, Kinzler KW and Vogelstein B (1993) *WAF1*, a potential mediator of p53 tumor suppression. *Cell* **75**: 817–825

Friedman LS, Ostermeyer EA, Szabo CI, Dowd P, Lynch, ED, Rowell SE and King MC (1994) Confirmation of BRCA1 by analysis of germline mutations linked to breast and ovarian cancer in ten families. *Nature Genet* **8**: 399–404

Gu Y, Turck CW and Morgan DO (1993) Inhibition of CDK2 activity *in vivo* by an associated 20K regulatory subunit. *Nature* **366**: 707–710

Guan K-L, Jenkins CW, Li Y, Nichols MA, Wu X, O'Keefe CL, Matera AG and Xiong Y (1994) Growth suppression by *p18*, a $p16^{INK4/MTS1}$ and $p14^{INK4B/MTS2}$ related CDK6 inhibitor, correlates with wild-type pRb function. *Genes Dev* **8**: 2939–2952

Hannon GJ and Beach D (1994) $P15^{INK4B}$ is a potential effector of TGF-β-induced cell cycle arrest. *Nature* **371**: 257–261

Herrington CS (1995) Human papillomaviruses and cervical neoplasia. II. Interaction of HPV with other factors. *J Clin Pathol* **48**: 1–6

Hirama T, Miller CW and Wilczynski S (1996) *p16* (CDKN2/Cyclin-dependent kinase-4 inhibitor/multiple tumor suppressor-1) gene is not altered in uterine cervical carcinomas or cell lines. *Modern Pathol* **9**: 26–31

Kamb A (1995) Cell-cycle regulators and cancer. *Trends Genet* **11**: 136–140

Kamb A, Gruis NA, Weaver-Feldhaus J, Liu Q, Harshman K, Tavtigian SV, Stockert E, Day III RS, Johnson BE and Skolnick MH (1994) A cell cycle regulator potentially involved in genesis of many tumor types. *Science* **264**: 436–440

Kelley MJ, Otterson GA, Kaye FJ, Popescu NC, Johnson BE and DiPaolo JA (1995) *CDKN2* in HPV-positive and HPV-negative cervical carcinoma cell lines. *Int J Cancer* **63**: 226–230

Koffa L, Koumantakis E, Ergazaki M, Tsatsanis C and Spandidos A (1995) Association of herpesvirus infection with the development of genital cancer. *Int J Cancer* **63**: 58–62

Li Y, Nichols MA, Shay JW and Xiong Y (1994) Transcriptional repression of the D-type cyclin-dependent kinase inhibitor p16 by the retinoblastoma susceptibility gene product pRb. *Cancer Res* **54**: 6078–6082

Liu Q, Neuhausen S, McClure M, Frye C, Weaver-Feldhaus J, Gruis NA, Eddington K, Allalunis-Turner MJ, Skolnick MH, Fujimura FK and Kamb A (1995) CDKN2 (MTS1) tumor suppressor gene mutations in human tumor cell lines. *Oncogene* **10**: 1061–1067

Lukas J, Parry D, Aagaard L, Mann DJ, Bartkova J, Strauss M, Peters G and Bartek J (1995) Retinoblastoma-protein-dependent cell-cycle inhibition by the tumor suppressor p16. *Nature* **375**: 503–506

Mitra AB, Murty VVVS, Li RG, Pratap M, Luthra UK and Chaganti RSK (1994) Allelotype analysis of cervical carcinoma. *Cancer Res* **54**: 4481–4487

Mullokandov MR, Kholodilov NG, Atkin NB, Burk RD, Johnson AB and Klinger HP (1996) Genomic alterations in cervical carcinoma: losses of chromosome heterozygosity and human papilloma virus tumor status. *Cancer Res* **56**: 197–205

Nakamura S, Akazawa K, Kinukawa N, Yao T and Tsuneyoshi M (1996) Inverse correlation between the expression of bcl-2 and p53 proteins in primary gastric lymphoma. *Human Pathol* **27**: 225–233

Nakao Y, Yang X, Yokoyama M, Pater MM and Pater A (1996) Malignant transformation of human ectocervical cells immortalized by HPV 18: in vitro model of carcinogenesis by cigarette smoke. *Carcinogenesis* **17**: 577–583

Noda A, Ning Y, Venable SF, Pereira-Smith OM and Smith JR (1994) Cloning of senescent cell-derived inhibitors of DNA synthesis using an expression screen. *Exp Cell Res* **211**: 90–98

Otterson GA, Kratzke RA, Coxon A, Kim YW and Kaye FJ (1994) Absence of $p16^{INK4}$ protein is restricted to the subset of lung cancer lines that retains wildtype RB. *Oncogene* **9**: 3375–3378

Pardee AB (1989) $G_1$ events and regulation of cell proliferation. *Science* **246**: 603–608

Polyak K, Lee M-H, Erdjument-Bromage H, Koff A, Roberts JM, Tempst P and Massagué J (1994) Cloning of $p27^{Kip1}$, a cyclin-dependent kinase inhibitor and a potential mediator of extracellular antimitogenic signals. *Cell* **78**: 59–66

Quesnel B, Fenaux P, Philippe N, Fournier J, Bonneterre J, Preudhomme C and Peyrat JP (1995) Analysis of p16 gene deletion and point mutation in breast carcinoma. *Br J Cancer* **72**: 351–353

Rossi AM and Hirschhorn RR (1991) Expression of growth-regulated genes in normal and SV40 transformed hamster fibroblasts. *J Cell Biochem* **47**: 165–173

Sakaguchi M, Fujii Y, Hirabayashi H, Yoon H-E, Komoto Y, Oue T, Kusafuka T, Okada A and Matsuda H (1996) Inversely correlated expression of p16 and Rb protein in non-small cell lung cancers: an immunohistochemical study. *Int J Cancer* **65**: 442–445

Sarma D, Yang X, Jin G, Shindoh M, Pater MM and Pater A (1996) Resistance to retinoic acid and altered cytokeratin expression of human papillomavirus type 16-immortalized endocervical cells after tumorigenesis. *Int J Cancer* **65**: 345–350

Spruck CH, Gonzalez-Zulueta M, Shibata A, Simoneau AR, Lin M-F, Gonzales F, Tsai YC and Jones PA (1994) P16 gene in uncultured tumors. *Nature* **370**: 183–184

Sreekantaiah C, Bhargava MK and Shetty NJ (1988) Chromosome 1 abnormalities in cervical carcinoma. *Cancer* **62**: 1317–1324

Sun Q, Tsutsumi K, Kelleher MB, Pater A and Pater MM (1992) Squamous metaplasia of normal and carcinoma *in situ* of HPV 16-immortalized human endocervical cells. *Cancer Res* **52**: 4254–4260

Sun Q, Tsutsumi K, Yokoyama M, Pater MM and Pater A (1993) *In vivo* cytokeratin-expression pattern of stratified squamous epithelium from human papillomavirus-type-16-immortalized ectocervical and foreskin keratinocytes. *Int J Cancer* **54**: 656–662

Tam SW, Shay JW and Pagano M (1994) Differential expression and cell cycle regulation of the cyclin-dependent kinase 4 inhibitor $p16^{Ink4}$. *Cancer Res* **54**: 5816–5820

Toyoshima H and Hunter T (1994) p27, a novel inhibitor of G1 cyclin-cdk protein kinase activity, is related to p21. *Cell* **78**: 67–74

Tsutsumi K, Belaguli N, Sun Q, Michalak TI, Gulliver WP, Pater A and Pater MM (1992) Human papillomavirus 16 DNA immortalizes two types of normal human epithelial cells of the uterine cervix. *Am J Pathol* **140**: 255–261

Vogelstein B and Kinzler KW (1993) The multistep nature of cancer. *Trends Genet* **9**: 138–141

von Knebel-Doeberitz M, Oltersdorf T, Schwarz E and Gissmann L (1988) Correlation to modified human papilloma virus early gene expression with altered growth properties in C4-I cervical carcinoma cells. *Cancer Res* **48**: 3780–3786

Weinberg RA (1995) The retinoblastoma protein and cell cycle control. *Cell* **81**: 323–330

Whitaker NJ, Bryan TM, Bonnefin P, Chang AC-M, Musgrove EA, Braithwaite AW and Reddel RR (1995) Involvement of RB-1, p53, $p16^{INK4}$ and telomerase in immortalization of human cells. *Oncogene* **11**: 971–976

Xiong Y, Hannon GJ, Zhang H, Casso D, Kobayashi R and Beach D (1993) p21 is a universal inhibitor of cyclin kinases. *Nature* **366**: 701–704

Yang X, Jin G, Nakao Y, Rahimtula M, Pater MM and Pater A (1996*a*) Malignant transformation of HPV16-immortalized human endocervical cells by cigarette smoke condensate and characterization of multistage carcinogenesis. *Int J Cancer* **65**: 338–344

Yang X, Nakao Y, Pater MM and Pater A (1996*b*) Identification of two novel cellular genes associated with multistage carcinogenesis of human endocervical cells by mRNA differential display. *Carcinogenesis* **17**: 563–567

Yeager T, Stadler W, Belair C, Puthenveettil J, Olopade O and Reznikoff C (1995) Increased p16 levels correlate with pRb alterations in human urothelial cells. *Cancer Res* **55**: 493–497

Yokota J, Tsukada Y, Nakajima T, Gotoh M, Shimosato Y, Mori N, Tsunokawa Y, Sugimura T and Terada M (1989) Loss of heterozygosity on the short arm of chromosome 3 in carcinoma of the uterine cervix. *Cancer Res* **49**: 3598–3601

Yokoyama M, Tsutsumi K, Pater A and Pater MM (1994) Human papillomavirus 18-immortalized endocervical cells with *in vitro* cytokeratin expression characteristics of adenocarcinoma. *Obstet Gynecol* **83**: 197–204

Yoshikawa H, Kawana T, Kitagawa K, Mizuno M, Yoshikura H and Iwamoto A (1991) Detection and typing of multiple genital human papillomaviruses by DNA amplification with consensus primers. *Jpn J Cancer Res* **82**: 524–531

Zhang S-Y, Klein-Szanto AJP, Sauter ER, Shafarenko M, Mitsunaga S, Nobori T, Carson DA, Ridge JA and Goodrow TL (1994) Higher frequency of alterations in the p16/*CDKN2* gene in squamous cell carcinoma cell lines than in primary tumors of the head and neck. *Cancer Res* **54**: 5050–5053

zur Hausen H (1994) Molecular pathogenesis of cancer of the cervix and its causation by specific human papillomavirus types. In *Current Topics Microbiology and Immunology: Human Pathogenic Papillomaviruses*, Vol. 186, zur Hausen H (ed.), pp. 131–156. Springer: Berlin zur Hausen H and de Villiers E-M (1994) Human papilloma viruses. *Annu Rev Microbiol* **48**: 427–447

*© Cancer Research Campaign 1997*